# Conjoint Analysis of Sheep Microbiome, Metabolome, and Transcriptome Revealed the Effect Mechanisms of Feeding with Broccoli Extract

**DOI:** 10.3390/vetsci13070663

**Published:** 2026-07-08

**Authors:** Gang Zhou, Ying Liu, Xuanxuan Pu, Qiugui Ning, Xiaoshan Guo, Liwei Wang, Yuhong Zhong, Guolian Wang, Xuefeng Guo, Mengzhi Wang

**Affiliations:** 1 College of Life Science and Technology, Tarim University, Alar 843300, China; 2 Xinjiang Production & Construction Corps Key Laboratory of Protection and Utilization of Biological Resources in Tarim Basin, Alar 843300, China; 3 Huaiyin Institute of Agricultural Sciences in Xuhuai Regio, Huaian 223000, China; 4 School of Life Sciences, Huaiyin Normal University, Huai’an 223300, China; 5 College of Animal Science and Technology, Tarim University, Alar 843300, China; 6 Key Laboratory of Livestock and Grass Resources Utilization around Tarim, Ministry of Agriculture and Rural Areas (Co-Construction by Ministries and Provinces), Alar 843300, China; 7 College of Animal Science and Technology, Yangzhou University, Yangzhou 225009, China

**Keywords:** sheep, broccoli tails extract, multi-omics, intestinal tract

## Abstract

Alterations in microbiota, transcript and metabolites are critical to intestinal homeostasis and host health. This study used a combination of 16s rRNA, transcriptome sequencing and liquid chromatography–mass spectrometry to investigate intestinal microbiota, genes and metabolic profiles in the ileum of Hu sheep fed broccoli extract. Here, we randomly allocated 14 Hu sheep to two diets: a basal diet without any supplementation (NC) and a basal diet supplemented with 200 mg/kg broccoli tail (BT). After 60 days of treatment, blood and jejunal samples were collected for serum biochemical indicators and multi-omics analysis. In this study, the extract of broccoli tails had a significant effect on the serum biochemical indicators, including white blood cells, red blood cells, mean corpuscular volume, mean corpuscular hemoglobin concentration, mean platelet volume, triglycerides and total protein in Hu sheep (*p* < 0.05). Transcriptomic analysis showed that the 672 differentially expressed genes between the NC and BT groups were primarily enriched in linoleic acid metabolism, steroid hormone biosynthesis, and cholesterol metabolism. Metabolomics analysis using Kyoto Encyclopedia of Genes and Genomes enrichment showed that the 41 differentially abundant metabolites were mainly enriched in bile secretion, vitamin B6 metabolism, and the mTOR signaling pathway. 16S rRNA sequencing results indicated that the extract of broccoli tails increased the relative abundance of *Peptostreptococcaceae* and decreased the relative abundance of Lachnospiraceae, Lachnospirales, and Bacteroidaceae. Integrated transcriptome, metabolome, and microbiome analysis showed that the gut microbiota and host transcriptomic changes may participate in systemic metabolic regulation by modulating amino acid metabolism, lipid signal transduction, nucleotide metabolism, and vitamin B6-related metabolic pathways. These findings demonstrate that the extract of broccoli tails modulates intestinal gene expression, systemic metabolism, and gut microbial ecology in Hu sheep, providing new insights into the utilization of agricultural byproducts as a functional feed supplement for ruminants.

## Simple Summary

Broccoli is a widely cultivated vegetable, but its stems and leaves are often discarded as agricultural waste. In this study, we found that broccoli extract altered the expression of hundreds of genes, altered metabolites involved in fat metabolism and vitamin processing, and shifted the gut bacteria composition toward a more diverse community in the small intestine of sheep. These changes suggest that broccoli tail extract promotes intestinal health and metabolic balance in sheep and supports the recycling of broccoli byproducts as a functional feed supplement, which could reduce agricultural waste while improving livestock productivity.

## 1. Introduction

The sustainable utilization of agricultural byproducts has become a priority in livestock production, driven by economic and environmental considerations [1]. Broccoli (*Brassica oleracea var. italica*) is one of the most widely cultivated vegetables worldwide, yet a substantial proportion of the plant—including stems, leaves, and floret residues—is discarded during harvesting and processing [2]. These byproducts, collectively referred to as broccoli “tails” or “waste,” contain various bioactive compounds, including glucosinolates, sulforaphane, flavonoids, vitamins (particularly vitamin C and vitamin B6), and dietary fiber [3,4]. Among these, sulforaphane, an isothiocyanate derived from the hydrolysis of glucoraphanin, has attracted considerable attention for its antioxidant, anti-inflammatory, and lipid metabolism-modulating properties [5]. In ruminant nutrition, the inclusion of plant-derived feed supplements has been explored as a strategy to improve animal health, feed efficiency, and product quality, while reducing the reliance on antibiotic growth promoters [6]. Previous studies have demonstrated that broccoli byproducts can be incorporated into ruminant diets without adverse effects. Broccoli byproduct–wheat straw silage may be fed to Fashandy lambs up to 200 g/kg of diet DM without any adverse impacts on growth performance [7]. In dairy ewes, supplementation with 1.5 kg of broccoli or cauliflower in lactating sheep did not affect feed intake, milk production or health status of the animals and had little impact on the quality of milk [8]. A recent study reported that supplementing daily with 15 g of broccoli extract for 45 days modulated the rumen microbiota composition and improved carcass traits in Holstein steers [9]. These findings collectively suggest that broccoli-derived compounds exert measurable biological effects in ruminants, yet the underlying molecular mechanisms remain incompletely understood.

The gastrointestinal tract of ruminants hosts a complex microbial ecosystem that plays a fundamental role in nutrient digestion, immune regulation, and host metabolism [10]. In sheep, the small intestine—particularly the jejunum—is a major site of nutrient absorption and mucosal immunity [11]. Disruptions to the intestinal transcriptome, metabolome, or microbial community can compromise barrier function and metabolic homeostasis [12]. Multi-omics approaches, integrating transcriptomic, metabolomic, and microbiome data, offer a powerful framework for comprehensively characterizing the biological responses to dietary interventions [13]. Such integrative analyses have been successfully applied to investigate rumen adaptation in Tibetan sheep [14], probiotic effects in fattening lambs [15], and host–microbe interactions in *E. coli*-challenged sheep [16], yet no study has employed this approach to evaluate the effects of broccoli-derived supplements on the sheep intestine.

Hu sheep, a prolific breed native to the Taihu Lake region of China, are of major economic importance in Chinese animal husbandry due to their high reproductive rate and rapid growth [17]. Optimizing the intestinal health of Hu sheep through dietary strategies represents a practical approach to enhancing their production performance and animal welfare. Broccoli is a nutritious vegetable that possesses antioxidant activities and is beneficial to the human body. However, the effect and mechanism of broccoli tail extract on the intestinal health of Hu sheep remain unclear, and multi-omics integration would reveal coordinated biological responses across these layers. The objective of this study was to systematically evaluate the effects of broccoli tail vegetable extract supplementation on the jejunal transcriptome, metabolome, and gut microbiome of Hu sheep using an integrated multi-omics approach and to construct gene–metabolite and microbe–metabolite interaction networks to elucidate the molecular mechanisms underlying the observed effects.

## 2. Materials and Methods

### 2.1. Experimental Design

The animal study protocol was approved by the Yangzhou University Animal Care and Use Committee of Jiangsu Province (China). Approval Code: SYXK (Su) 2016-0019. Approval Date: 6 April 2016.

All experimental animals were sourced from Yeqing Goat Farm, Lianshui County, Huai’an City, Jiangsu Province, China, and were housed in an independent sheep barn on the same farm. Broccoli tail extract (1% concentration sulforaphane) was purchased from Ciyuan Biotechnology Co., Ltd. (Baoji, Shanxi, China). A total of 14 five-month-old Hu sheep rams with an initial body weight of 30 ± 3 kg were selected and randomly assigned to two groups of seven animals each. The control group (NC) received a basal diet without any supplementation throughout the 60-day experimental period, while the treatment group (BT) received the basal diet supplemented with broccoli tail vegetable extract from day 1 to 60. The broccoli tail extract was administered at a dose of 200 mg/kg body weight (BW) per day. The broccoli tail extract was prepared from fresh broccoli root through aqueous extraction and lyophilization. All animals were fed the same basal diet formulated to meet the nutritional requirements of growing Hu sheep. Prior to the commencement of the experiment, the sheep barn was thoroughly cleaned, disinfected, and partitioned into individual pens (1.2 m × 1.5 m), each equipped with a drinking water dispenser. Throughout the experimental period, all Hu sheep had unrestricted access to both feed and water. The ambient temperature was maintained within the thermoneutral zone for sheep.

### 2.2. Sample Collection

At the end of the 60-day experimental period, all sheep were fasted for 12 h and then humanely slaughtered in accordance with standard commercial procedures at a licensed abattoir. Blood samples from the control (n = 7) and treatment groups (n = 7) were collected from the jugular vein immediately before slaughter using disposable syringes aseptically. The collected blood was transferred into 10 mL Eppendorf tubes containing 100 μL of 0.5 M ethylenediaminetetraacetic acid (EDTA) as an anticoagulant. The preserved blood samples were transported to the laboratory on ice and stored at −20 °C for subsequent analyses.

Following slaughter, digesta samples from the mid-jejunum were collected and snap-frozen in liquid nitrogen for 16S rRNA sequencing. Mucosal epithelial tissues from the jejunum were dissected, immediately placed in liquid nitrogen, and stored at −80 °C for transcriptome and metabolome analyses.

### 2.3. Measurement of Serum Biochemical Indicators

Quantitative analysis of the hematological parameters, including white blood cells (WBCs), red blood cells (RBCs), hemoglobin (HGB), hematocrit (HCT), mean corpuscular volume (MCV), mean corpuscular hemoglobin concentration (MCHC), red cell distribution width–coefficient of variation (RDW-CV), platelets (PLT), and mean platelet volume (MPV), was performed using an automated hematology analyzer (BS-200, Mindray Bio-Medical Electronics Co., Ltd., Shenzhen, China). Serum biochemical parameters, including blood urea nitrogen (BUN), alanine transaminase (ALT), aspartate aminotransferase (AST), phosphorus (P), calcium (Ca), triglycerides (TG), and total protein (TP), were measured using an automated biochemical analyzer (Mindray, Shenzhen, China).

### 2.4. Transcriptome Analyses

Total RNA was isolated and purified from jejunal tissue samples using TRIzol reagent (Invitrogen, Carlsbad, CA, USA), according to the manufacturer’s instructions. RNA concentration and purity were assessed using a NanoDrop ND-1000 UV–Vis spectrophotometer (NanoDrop, Wilmington, DE, USA). RNA integrity was evaluated using a Bioanalyzer 2100 (Agilent Technologies, Santa Clara, CA, USA), with samples exhibiting an RNA integrity number (RIN) > 7.0 retained for library construction.

A total of 14 RNA-sequencing libraries from jejunal tissues of the control (n = 7, NC) and treatment groups (n = 7, BT) were constructed. Paired-end sequencing (150 bp) was performed on an Illumina NovaSeq platform at Novogene Bioinformatics Institute (Beijing, China). Raw FASTQ data were processed using Cutadapt to remove adapter sequences at the 3′ end, and reads with an average quality score below Q20 were filtered out. Clean reads were aligned to the sheep reference genome (ARS-UI_Ramb_v2.0) using HISAT2. Gene expression levels were calculated as fragments per kilobase per million mapped reads (FPKM). Differentially expressed genes (DEGs) between the NC and BT groups were identified using edgeR, with |log2 fold change| > 1 and *p* < 0.05 as the screening criteria. Gene Ontology (GO) and Kyoto Encyclopedia of Genes and Genomes (KEGG) pathway enrichment analyses of DEGs were performed using the clusterProfiler package (version 4.6.2) in R [18].

### 2.5. Metabolome Profile Analyses

Jejunum metabolome profiling was performed using ultra-high-performance liquid chromatography (UHPLC) coupled with a Q-Exactive high-resolution mass spectrometer (Thermo Fisher Scientific Inc., Waltham, MA, USA) in both positive and negative ionization modes. Chromatographic separation was achieved using a C18 column (2.1 mm × 100 mm, 1.8 μm) with a gradient elution system. The raw mass spectrometry data were processed using the Compound Discoverer 3.0 software (Thermo Fisher Scientific Inc.) for peak extraction, alignment, correction, normalization, and filtering. Metabolite structures were identified through accurate mass matching (<25 ppm) and MS/MS spectral matching against public databases. Principal component analysis (PCA) and orthogonal partial least squares discriminant analysis (OPLS-DA) were performed using MetaboAnalyst 5.0 (https://www.metaboanalyst.ca/, accessed on 5 December 2025) [19]. Differentially abundant metabolites (DAMs) were screened using the criteria of variable importance in projection (VIP) > 1 and *p* < 0.05. Metabolite classification and KEGG pathway enrichment analysis were conducted using the MetOrigin platform (http://metorigin.met-bioinformatics.cn/app/metorigin, accessed on 20 December 2025) [20].

### 2.6. Sequencing Analysis of 16S rRNA

Microbial genomic DNA was extracted from jejunal digesta samples using the QIAamp DNA Stool Mini Kit (Qiagen, Hilden, Germany), according to the manufacturer’s instructions. DNA integrity and purity were assessed using 1% agarose gel electrophoresis and a NanoDrop 8000 spectrophotometer (Thermo Fisher Scientific Inc.). Sequencing libraries targeting the V3-V4 hypervariable region of the bacterial 16S rRNA gene were prepared via PCR amplification using primers 341F (5′-ACTCCTACGGGRSGCAGCAG-3′) and 806R (5′-GGACTACVVGGGTATCTAATC-3′) [21], supplemented with Illumina sequencing adapters and sample-specific barcodes. All libraries were pooled and sequenced using an Illumina HiSeq Rapid SBS Kit V2 on the HiSeq 2500 platform (PE250 mode) at Novogene Bioinformatics Institute.

Quality filtering, trimming, denoising, and merging of FASTQ files were performed using the DADA2 package [22] in QIIME2 (version 2020.6.0) [23]. Amplicon sequence variants (ASVs) were generated, and taxonomic classification was performed against the SILVA database. Alpha diversity indices (Chao1, Shannon, and Simpson) were calculated and compared between groups using Wilcoxon rank-sum tests. Beta diversity was assessed using principal coordinates analysis (PCoA) based on Bray–Curtis distances. Linear discriminant analysis effect size (LEfSe) was used to identify biomarkers with statistically significant differences between groups (LDA score threshold = 4.0). The functional prediction of the gut microbiota was conducted using PICRUSt2 [24].

### 2.7. Correlation Analysis of 16S rRNA, Transcriptome, and Metabolome

Correlations between differentially abundant microbial genera, differentially expressed genes, and differentially abundant metabolites were estimated using Spearman correlation analysis with the ‘pheatmap’ package in R (version 3.3.1). Multi-omics integration analyses were performed using the mixOmics package [25,26] in R to construct gene–metabolite and microbe–metabolite correlation networks.

### 2.8. Data Analyses

Differences in non-parametric data between the two groups were analyzed using the Wilcoxon rank-sum test. For all other data, Student’s *t*-test was used to compare the differences between the NC and BT groups. Statistical analyses were performed using SPSS 20.0 software (SPSS, Inc., Chicago, IL, USA). All the values were expressed as mean ± standard deviation (SD), and a *p*-value < 0.05 was considered statistically significant.

## 3. Results

### 3.1. Blood and Serum Biochemical Profiles of Sheep Supplemented with Broccoli Extract

The dietary supplementation of broccoli exhibited a significant (*p* < 0.05) effect on the sheep blood profile (Table 1). A significantly (*p* < 0.05) higher count of WBC (9.57 × 10^9^ /L ± 1.57) was found in the normal diet group (NC). The highest count of RBC was found in blood from the broccoli tail extract-supplemented group (BT) (9.26 × 10^12^/L ± 0.56), compared to the sheep in the NC group (5.57 × 10^12^/L ± 0.74) (*p* < 0.01). A significantly (*p* < 0.01) higher MCV was found in the BT group (32.24 ± 2.42) compared to the NC group (27.52 ± 1.04). The BT group showed significantly (*p* < 0.01) higher MCHC value (338.28 ± 12.89) compared to the NC group (333.85 ± 9.29). A similar trend was found for MPV, with significantly (*p* < 0.01) higher MPV in the BT group (4.95 ± 0.35), compared to the NC group (4.41 ± 0.41). A non-significant (*p* > 0.05) variation was found in other blood parameters, such as HGB, HCT, RDW-CV, and PLT. The dietary supplementation of broccoli significantly (*p* < 0.05) increased the essential serum biochemical parameters (TP and TG) in sheep (Table 2). Significantly (*p* < 0.01) high TP (67.67 ± 3.05) and TG (0.48 ± 0.01) levels were found in the BT group, compared to the NC group. These findings indicate that broccoli likely had an impact on the improvement of sheep immunity.

### 3.2. Transcriptome Analysis of Sheep Supplemented with Broccoli Extract

**Figure 1:**
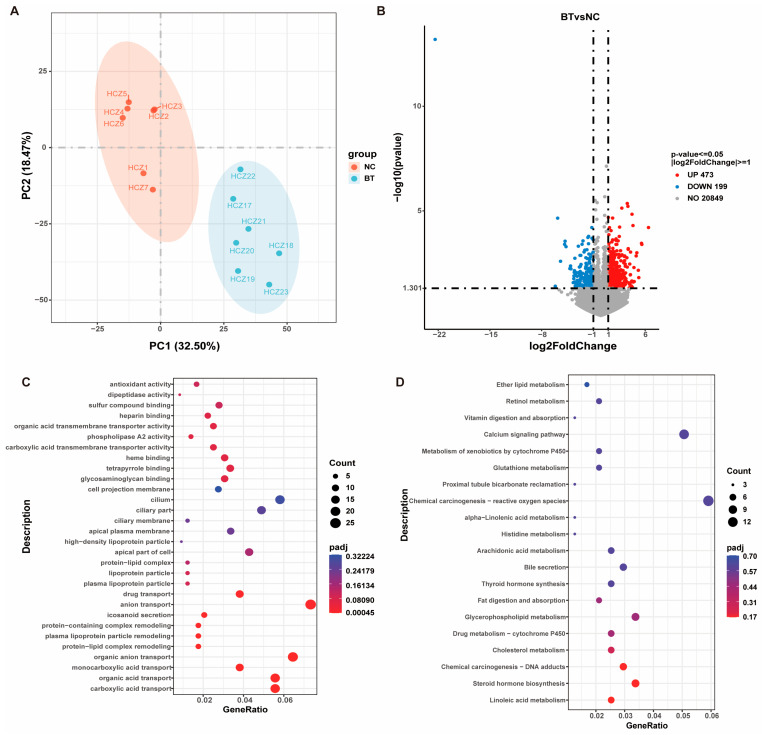
Transcriptomic analysis of sheep jejunal tissues between the BT and NC groups. (**A**) Principal component analysis (PCA) of all samples. NC, normal diet group; BT, broccoli tail extract-supplemented group. (**B**) Volcano plot displaying differentially expressed genes (DEGs) between the BT and NC groups. Red dots represent upregulated DEGs; blue dots represent downregulated DEGs. (**C**) GO enrichment histogram of DEGs. (**D**) KEGG enrichment scatter plot of DEGs. The size of the dot represents the level of DEG enrichment. The color of the dot represents the significance of DEG enrichment.

After quality filtering and control of the raw sequencing data, an average of 704,957,952 high-quality clean reads were obtained from the two groups. The Q30 base distribution ranged from 96.85% to 97.32%, with an average GC content of 51.66%. The overall sequencing error rate was 0.01%, and over 91% of reads in each sample aligned to the sheep reference genome (Table S1). PCA showed that the samples within each group exhibited relatively good biological replication (Figure 1A).

Based on the transcriptomic sequencing results, DEGs were screened using the criteria of |log2 fold change| ≥ 1 and FDR < 0.05. A total of 672 DEGs were identified between the BT and NC groups, of which 473 genes were upregulated, and 199 genes were downregulated in the BT group (Figure 1B, Table S2).

GO enrichment analysis revealed that the DEGs were primarily enriched in carboxylic acid transport (GO:0046942), organic acid transport (GO:0015849), organic anion transport (GO:0015711), and anion transport (GO:0006820) (Figure 1C). KEGG pathway enrichment analysis identified 20 significantly enriched signaling pathways in the BT vs. NC comparison (Figure 1D, Table S3). The most prominently enriched pathways included linoleic acid metabolism (oas00591), steroid hormone biosynthesis (oas00140), chemical carcinogenesis–DNA adducts (oas05204), and cholesterol metabolism (oas04979).

### 3.3. Metabolomic Analysis of Sheep Supplemented with Broccoli Extract

**Figure 2:**
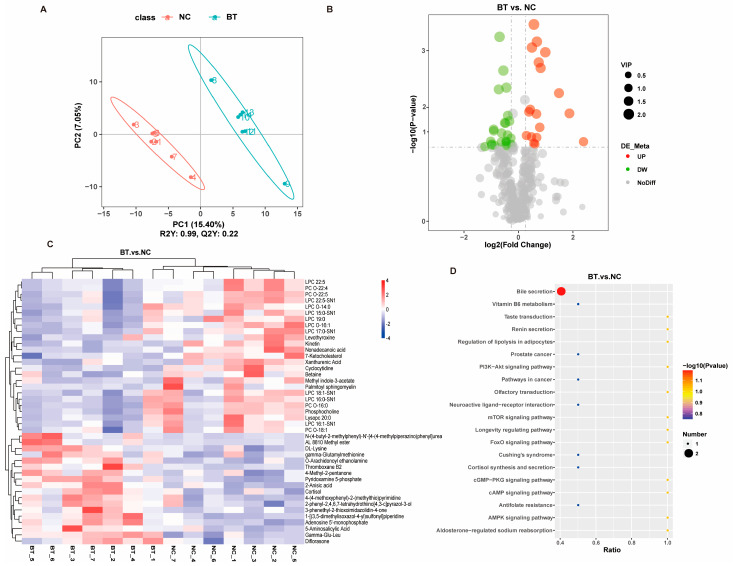
LC-MS/MS analysis of jejunum metabolic profiles between the BT and NC groups. (**A**) Volcano plot of differentially abundant metabolites between the BT and NC groups. NC, normal diet group; BT, broccoli tail extract-supplemented group. (**B**) OPLS-DA score plot. (**C**) Heatmap analysis of differentially abundant metabolites (DAMs) between the BT and NC groups. (**D**) KEGG enrichment scatter plot of DAMs.

The metabolomic analysis of the jejunum samples identified a total of 387 metabolites (Table S4). PCA revealed distinct metabolic profiles between the BT and NC groups. OPLS-DA showed that the R2X, R2Y, and Q2 values were close to 1 (Q2 > 0.9), confirming the reliability and predictive capacity of the model (Figure 2A). Using the screening criteria of VIP > 1 and *p* < 0.05, a total of 41 differentially abundant metabolites (DAMs) were identified, with 18 upregulated and 23 downregulated metabolites in the BT group (Figure 2B,C). Functional annotation and KEGG pathway enrichment analysis of the DAMs revealed significant enrichment in pathways including bile secretion, vitamin B6 metabolism, the mTOR signaling pathway, and the PI3K-Akt signaling pathway (Figure 2D).

### 3.4. Microbiome Analysis of Sheep Supplemented with Broccoli Extract

**Figure 3:**
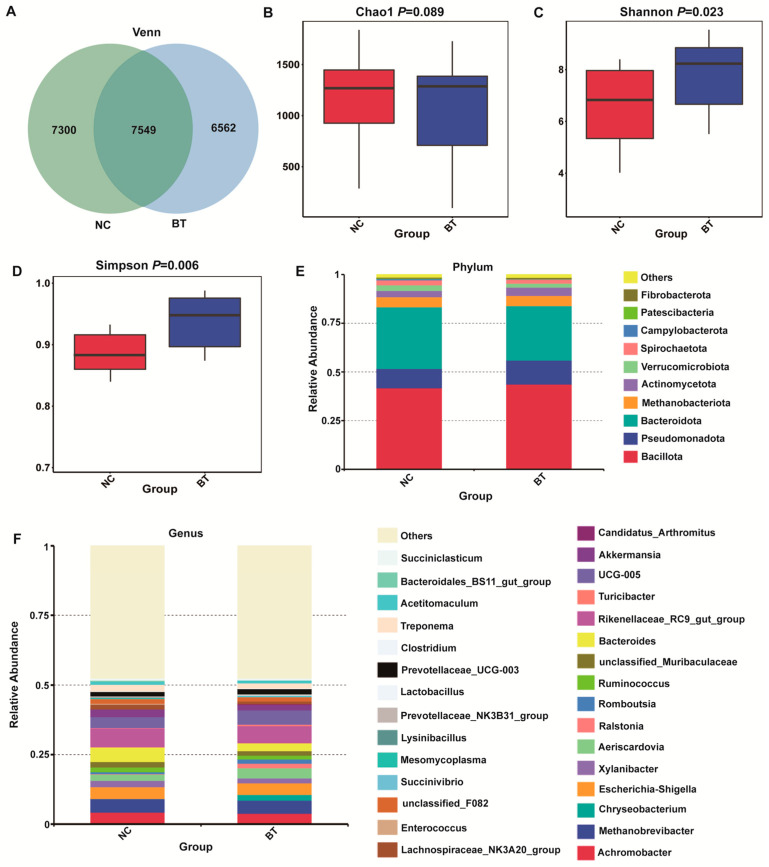
Analysis of microbial changes in the microbiome. (**A**) Venn plot of ASV between the BT and NC groups. ASV, Amplicon Sequence Variant; NC, normal diet group; BT, broccoli tail extract-supplemented group. (**B**–**D**) Bacterial alpha diversity determined by Chao1, Shannon, and Simpson indices, respectively. (**E**,**F**) Microbial distributions of the different groups at the phylum and genus levels.

To characterize the effects of the extract of broccoli tails on the jejunal microbiome, 16S rRNA gene sequencing was performed on jejunal digesta samples. The Venn diagram revealed 7300 amplicon sequence variants (ASVs) unique to the NC group, 6562 ASVs unique to the BT group, and 7549 shared ASVs, indicating substantial differences in bacterial species composition between the two groups (Figure 3A).

The alpha diversity analysis showed no significant difference in the Chao1 index between the two groups (*p* > 0.05, Figure 3B), indicating comparable species richness. However, the BT group exhibited a significantly higher Shannon index than the NC group (*p* < 0.05, Figure 3C), and the Simpson index in the BT group was significantly higher than in the NC group (*p* < 0.01, Figure 3D), indicating that BT supplementation enhanced microbial diversity.

PCoA based on Bray–Curtis distances showed a clear separation of jejunal microbiota composition between the BT and NC groups, with PC1 and PC2 contributing 51.48% and 14.91% of the total variation, respectively. Taxonomic analysis revealed that Bacillota, Bacteroidota, Pseudomonadota, Methanobacteriota, and Actinomycetota were the most abundant phyla in both groups, with Bacillota and Bacteroidota showing the highest relative abundance (Figure 3E). At the genus level, the most abundant taxa included Rikenellaceae RC9 gut group, UCG-005, Methanobrevibacter, Escherichia–Shigella, and Achromobacter (Figure 3F).

**Figure 4:**
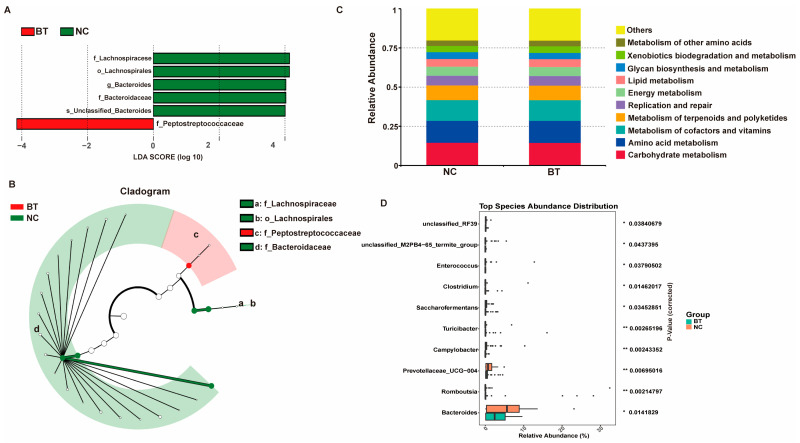
LEfSe analysis and PICRUSt functional prediction. (**A**,**B**) LEfSe results from the colonic microbiota indicating bacteria that are significantly associated with the BT and NC group samples (LDA score = 4). NC, normal diet group; BT, broccoli tail extract-supplemented group. (**C**) PICRUSt functions predicting the top 10 stacking maps at level 2. (**D**) Top species abundance distribution with significant differences between the BT and NC groups. * *p* < 0.05, ** *p* < 0.01.

LEfSe analysis identified six biomarkers at different taxonomic levels between the BT and NC groups (Figure 4A). Within the NC group, Lachnospiraceae (family), Lachnospirales (order), Bacteroides (genus), Bacteroidaceae (family), and unclassified Bacteroides (species) were significantly enriched. In contrast, Peptostreptococcaceae (family) was significantly enriched in the BT group. The cladogram (Figure 4B) indicated that the differentially abundant microorganisms in the NC group were primarily concentrated within Lachnospiraceae, Lachnospirales, and Bacteroidaceae, while the BT group was characterized by the enrichment of Peptostreptococcaceae.

PICRUSt2 functional prediction analysis (KEGG level 2) revealed that the gut microbiota were primarily involved in carbohydrate metabolism, amino acid metabolism, metabolism of terpenoids and polyketides, energy metabolism, and lipid metabolism (Figure 4C). Analysis of the top differentially abundant genera between the two groups revealed significantly different abundances of Bacteroides, Romboutsia, Prevotellaceae UCG-004, Campylobacter, Turicibacter, Saccharofermentans, Clostridium, Enterococcus, unclassified M2PB4-65 termite group, and unclassified RF39 (*p* < 0.05, Figure 4D).

### 3.5. Interaction Analysis of the Transcriptome and Metabolome in Sheep Supplemented with Broccoli Extract

**Figure 5:**
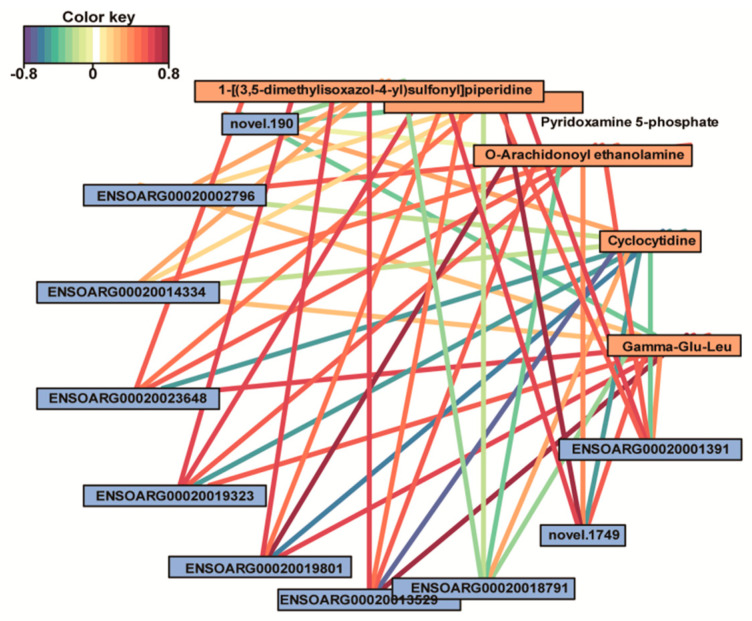
Transcriptome and metabolome correlation analysis. The top 5 metabolites from the metabolomic enrichment analysis and the top 10 genes from the transcriptomic analysis were selected. A metabolite–transcript correlation network was constructed and visualized using the mixOmics package in R. Rectangular nodes represent either metabolites (yellow) or genes (blue). The edge color denotes the direction of correlation: red lines indicate positive correlations, and blue lines indicate negative correlations. The line width and color intensity reflect the magnitude of the correlation coefficient.

Based on the integrated transcriptomic and metabolomic analysis, a correlation network between genes and metabolites was constructed using mixOmics (Figure 5). The network comprised 15 nodes in total, including 10 transcript nodes and 5 metabolite nodes. Several gene nodes, including ENSOARG00020002796, ENSOARG00020014334, ENSOARG00020023648, ENSOARG00020019323, ENSOARG00020019801, ENSOARG00020013529, ENSOARG00020018791, and ENSOARG00020001391, were connected to multiple metabolites, exhibiting characteristics of hub genes. Similarly, the metabolites pyridoxamine 5′-phosphate (associated with vitamin B6 metabolism), O-arachidonoyl ethanolamine (a lipid signaling molecule), and gamma-Glu-Leu (an amino acid metabolite) were each linked to multiple genes, suggesting central regulatory roles within the relevant metabolic pathways. Collectively, this network reveals molecular interactions between the transcriptional and metabolic levels, encompassing amino acid metabolism, lipid signal transduction, nucleoside metabolism, and vitamin B6 metabolism.

### 3.6. Interaction Analysis of Metabolome and Microbiome in Sheep Supplemented with Broccoli Extract

**Figure 6:**
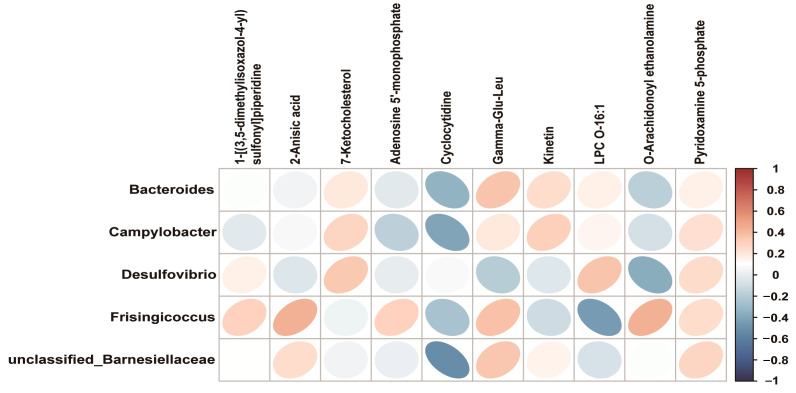
Microbiome and metabolome correlation analysis. The correlation heatmap illustrates pairwise correlations between differentially abundant bacterial genera and differentially abundant metabolites. The horizontal axis represents differential bacterial genera; the vertical axis represents differential metabolites. Warmer red colors denote stronger positive correlations; deeper blue colors indicate stronger negative correlations. Ellipse elongation reflects the absolute correlation value. Spearman’s rank correlation analysis was applied, and the resulting matrix was visualized using the corrplot package in R.

Based on the integrated microbiome and metabolome analysis, an ellipse correlation heatmap was generated to visualize the relationships between the five gut bacterial genera and ten metabolites (Figure 6). Bacteroides exhibited significant positive correlations with cyclocytidine, gamma-Glu-Leu, kinetin, LPC O-16:1, O-arachidonoyl ethanolamine, and pyridoxamine 5′-phosphate, while showing a moderate negative correlation with 2-anisic acid. Campylobacter displayed a marked positive correlation with 1-(3,5-dimethylisoxazol-4-yl)sulfonyl piperidine and a moderate positive correlation with 7-ketocholesterol, whereas its associations with most other metabolites were relatively weak. Desulfovibrio showed a moderate negative correlation with adenosine 5′-monophosphate and weak negative correlations with several other metabolites. Frisingicoccus demonstrated a strong negative correlation with 1-(3,5-dimethylisoxazol-4-yl)sulfonyl piperidine and moderate negative correlations with both 7-ketocholesterol and adenosine 5′-monophosphate. Among all genera examined, unclassified Barnesiellaceae exhibited the most pronounced negative correlations overall, with the strongest negative association observed for adenosine 5′-monophosphate. These findings demonstrate that distinct gut microbial genera are differentially associated with specific metabolites, suggesting that the gut microbiota may participate in systemic metabolic regulation by modulating amino acid metabolism, lipid signal transduction, nucleotide metabolism, and vitamin B6-related metabolic pathways.

## 4. Discussion

This study represents the first comprehensive multi-omics investigation of the effects of broccoli tail vegetable extract on the intestinal health of Hu sheep. By integrating transcriptomic, metabolomic, and microbiome analyses, we demonstrate that the extract of broccoli tails coordinately modulates jejunal gene expression, metabolite profiles, and the gut microbial ecology in these sheep, revealing interconnected molecular networks that may underlie the biological effects of broccoli-derived bioactive compounds in ruminants. The identification of 672 DEGs in jejunal tissues, with a predominance of upregulated genes (473/672), indicates that the extract of broccoli tails broadly activates transcriptional programs in the small intestine. The enrichment of DEGs in organic acid and anion transport GO terms suggests enhanced nutrient absorption capacity, which may reflect the metabolic demands of processing broccoli-derived compounds, consistent with previous observations of altered jejunal gene expression in response to dietary modulation in ruminants [28]. The KEGG pathway analysis revealed prominent enrichment in lipid metabolism-related pathways, particularly linoleic acid metabolism, steroid hormone biosynthesis, and cholesterol metabolism, consistent with the known effects of sulforaphane and other isothiocyanates on lipid metabolism in mammals [29,30]. Sulforaphane has been shown to modulate lipid metabolism by enhancing mitochondrial function and biogenesis, as well as activating nuclear factor E2-related factor 2 (Nrf2), a master regulator of antioxidant and detoxification responses [31]. The enrichment of the steroid hormone biosynthesis pathway suggests that broccoli-derived compounds may influence intestinal steroid metabolism, potentially affecting mucosal barrier function and local immune regulation. The cholesterol metabolism pathway enrichment aligns with recent findings that broccoli extract supplementation influenced lipid metabolism in fattening lambs [9], suggesting a conserved mechanism across ruminant species.

The identification of 41 DAMs in jejunal samples indicates that the extract of broccoli tails induces systemic metabolic changes beyond the intestinal tract. The enrichment of DAMs in bile secretion and vitamin B6 metabolism pathways is noteworthy. Bile acids play critical roles not only in lipid digestion and absorption but also in signaling through farnesoid X receptor (FXR) and G protein-coupled bile acid receptor 1 (GPBAR1/TGR5), which regulate glucose and lipid homeostasis [32]. The modulation of bile-related metabolites suggests that the extract of broccoli tails may influence bile acid metabolism, potentially through alterations in the gut microbial community, which is a major determinant of bile acid composition [33]. The enrichment of vitamin B6 metabolism is particularly relevant, given that broccoli is a rich source of pyridoxine and related vitamers [4]. Pyridoxamine 5′-phosphate, identified as a hub metabolite in the gene–metabolite network, participates in aminotransferase reactions and one-carbon metabolism, both of which are essential for intestinal epithelial cell proliferation and function [34]. The mTOR and PI3K-Akt signaling pathways enriched among the DAMs are central regulators of cell growth, proliferation, and survival [35], and their modulation by the extract of broccoli tails may reflect enhanced intestinal epithelial turnover and mucosal maintenance.

The extract of broccoli tails significantly increased the Shannon and Simpson diversity indices without altering the Chao1 richness estimator, indicating that the extract enhanced microbial evenness rather than species richness. Increased microbial diversity is generally associated with better ecosystem stability and resilience [36] and has been linked to improved gut health in ruminants [37]. The phylum-level composition, dominated by Bacillota and Bacteroidota, is consistent with the typical intestinal microbiome of sheep [38]. LEfSe analysis revealed that Peptostreptococcaceae was significantly enriched in the BT group, while Lachnospiraceae, Lachnospirales, and Bacteroidaceae were enriched in the NC group. Peptostreptococcaceae includes several taxa known for their involvement in protein fermentation and amino acid metabolism [39], which may relate to the protein-rich nature of broccoli byproducts. The reduction in Lachnospiraceae and Bacteroidaceae in the BT group is noteworthy, as certain members of these families are associated with carbohydrate fermentation and butyrate production [40]. The shift in microbial community structure observed in this study is consistent with the findings of a recent report that broccoli extract supplementation modulated rumen microbiota in lambs [9], although the specific taxa affected differed between the rumen and jejunum, reflecting the distinct ecological niches of these gastrointestinal segments.

The gene–metabolite correlation network constructed via mixOmics analysis identified hub genes and metabolites connected across multiple metabolic pathways, including amino acid metabolism, lipid signal transduction, nucleoside metabolism, and vitamin B6 metabolism. The identification of pyridoxamine 5′-phosphate, O-arachidonoyl ethanolamine, and gamma-Glu-Leu as hub metabolites, each linked to multiple genes, suggests that the extract of broccoli tails exerts pleiotropic effects on intestinal metabolism. Pyridoxamine 5′-phosphate, as a vitamin B6 derivative, may serve as a molecular bridge between dietary broccoli components and host metabolic regulation. O-arachidonoyl ethanolamine, an endocannabinoid-like lipid mediator, has been implicated in intestinal inflammation resolution and mucosal barrier maintenance [41]. The microbe–metabolite correlation analysis revealed that Bacteroides was positively correlated with multiple metabolites, including pyridoxamine 5′-phosphate and O-arachidonoyl ethanolamine, while Frisingicoccus and unclassified Barnesiellaceae showed predominantly negative correlations. These associations suggest that specific gut bacteria may mediate the metabolic effects of the extract of broccoli tails, either through direct metabolite production or through modulation of host metabolic pathways. The differential microbe–metabolite associations observed here are consistent with the concept of functional redundancy in the gut microbiome, where different taxa contribute to overlapping metabolic outputs [42]. Thus, the present study provides valuable evidence supporting the potential of broccoli tail extract as a functional feed supplement for Hu sheep. The coordinated modulation of gene expression, metabolite profiles, and microbial ecology suggests that broccoli byproducts may promote intestinal health through multiple, potentially synergistic, mechanisms. However, this study initially revealed the candidate genes, metabolites, and microbiota related to the influence of broccoli extract on the intestinal health of Hu sheep by using multi-omics. In the future, it is necessary to explore the function and mechanisms of these molecules at the cellular level. In addition, we will screen the concentration of broccoli extract, expand the sample size of Hu sheep, and complete the determination of production performance, which will further verify the feasibility of using broccoli tail extract as a functional feed supplement for Hu sheep.

## 5. Conclusions

In summary, this study demonstrated that supplementation with broccoli tail vegetable extract (200 mg/kg BW/day) modulated the jejunal gene expression, metabolite profiles, and gut microbial ecology in Hu sheep. Transcriptomic analysis identified 672 DEGs enriched in lipid metabolism and nutrient transport pathways. Metabolomic profiling revealed 41 DAMs associated with bile secretion, vitamin B6 metabolism, and cell signaling pathways. Microbiome analysis showed that the extract of broccoli tails enhanced microbial diversity and shifted the community structure, with enrichment of Peptostreptococcaceae. Multi-omics integration enabled the construction of gene–metabolite and microbe–metabolite interaction networks, with hub nodes linked to amino acid metabolism, lipid signaling, and vitamin B6 pathways. These findings provide new insights into the molecular mechanisms by which broccoli tail extract may contribute to improved intestinal health and metabolic regulation and support the potential development of broccoli tail extract as a sustainable functional feed supplement for sheep production. Future studies should focus on dose optimization, the functional validation of hub genes and metabolites, and the evaluation of production performance outcomes.

## Figures and Tables

**Table 1:** Blood profile analyses of sheep between the NC and BT groups.

Group	WBC (×10^9^/L)	RBC (×10^12^/L)	HGB (g/L)	HCT (%)	MCV (fL)	MCHC (g/L)	RDW-CV (%)	PLT (×10^9^/L)	MPV (fL)
NC	9.57 ± 1.57	5.57 ± 0.74	100.28 ± 3.25	27.07 ± 2.22	27.52 ± 1.04	333.85 ± 9.29	14.20 ± 0.51	507.71 ± 27.65	4.41 ± 0.41
BT	7.05 ± 1.14	9.26 ± 0.56	100.42 ± 3.35	26.91 ± 3.15	32.24 ± 2.42	338.28 ± 12.89	14.92 ± 0.96	508.28 ± 42.36	4.95 ± 0.35
RV	5.0–14.0	7.8–13.8	90–155	26–45	25–38	320–380	13–18	180–680	3.8–6.0
Sig.	*	**	NS	NS	**	**	NS	NS	**

NC, normal diet group; BT, broccoli tail extract-supplemented group; RV, reference value (normal range) [27]; WBCs, white blood cells; RBCs, red blood cells; HGB, hemoglobin; HCT, hematocrit; MCV, mean corpuscular volume; MCHC, mean corpuscular hemoglobin concentration; RDW-CV, red cell distribution width–coefficient of variation; PLT, platelets; MPV, mean platelet volume; * *p* < 0.05, ** *p* < 0.01, NS *p* > 0.05.

**Table 2:** Serum biochemistry analyses of sheep between the NC and BT groups.

Group	TP (g/L)	AST (U/L)	ALT (U/L)	BUN (mmol/L)	TG (mmol/L)	Ca (mmol/L)	P (mmol/L)
NC	60.44 ± 1.24	79.57 ± 3.10	29.85 ± 2.41	7.60 ± 0.31	0.37 ± 0.02	2.42 ± 0.08	2.21 ± 0.20
BT	67.67 ± 3.05	79.42 ± 2.99	29.71 ± 2.69	7.27 ± 0.44	0.48 ± 0.01	2.43 ± 0.08	2.11 ± 0.11
RV	56.0–78.0	40–96	0–42	2.5–10.4	0.3–0.54	2.28–2.70	1.29–2.87
Sig.	**	NS	NS	NS	**	NS	NS

NC, normal diet group; BT, broccoli tail extract-supplemented group; RV, reference value (normal range) [27]; TP, total protein; AST, aspartate aminotransferase; ALT, alanine transaminase; BUN, blood urea nitrogen; TG, triglycerides; ** *p* < 0.01, NS *p* > 0.05.

## Data Availability

The original contributions presented in this study are included in the article/Supplementary Material. Further inquiries can be directed to the corresponding authors.

## Supplementary Materials

The following supporting information can be downloaded at https://www.mdpi.com/article/10.3390/vetsci13070663/s1, Table S1: Statistics for the transcriptome sequencing data; Table S2: Differentially expressed genes of jejunal tissues between BT and NC groups in sheep; Table S3: List of KEGG pathways obtained via enrichment analysis; Table S4: Identification of metabolites in sheep jejunum based on LC-MS/MS analysis.
